# Co-Design of an Educational Resource with Female Partners of Male Stroke Survivors to Support Physical Activity Participation

**DOI:** 10.3390/ijerph192416856

**Published:** 2022-12-15

**Authors:** Allyson Calder, Gisela Sole, Hilda Mulligan

**Affiliations:** Centre for Health, Activity, and Rehabilitation Research, School of Physiotherapy, University of Otago, Dunedin 9054, New Zealand

**Keywords:** co-design, support persons, stroke, men

## Abstract

Many male stroke survivors find it challenging to meet the recommended physical activity (PA) guidelines for health benefits. The spouse/partner is an important source of self-management for stroke survivor PA participation; however, they feel unsupported by health professionals. This study aimed to co-design an educational resource prototype to guide and empower female partners in supporting male stroke survivors’ participation in PA. We used a participatory action research (PAR) methodology. Thirteen support persons of male stroke survivors from Canterbury, New Zealand participated in four PAR cycles. The data were collected using individual interviews and focus groups and analyzed inductively using the general inductive approach. Three themes were reflected in the data and informed the prototype content: (1) managing an unwanted and challenging new life, (2) inconsistent access to meaningful information, and (3) considerations for successful stroke survivor PA participation. If partners are to be an essential source in supporting stroke survivors’ self-management of PA, they require resources that are meaningful and credible to enhance their confidence and self-efficacy. Further research is needed to explore the acceptability and usability of the educational resource with a wider audience and evaluate the co-design process. An inclusive and collaborative approach where support persons were valued for their expertise was essential in co-designing a meaningful resource intended to support stroke survivors and support persons’ self-management of their PA.

## 1. Introduction

Stroke ranks in the top five leading causes of disability worldwide [1]. The incidence of a stroke is higher in men due to experiencing the onset of a stroke at an earlier age [2]. Regular moderate intensity physical activity (PA) is important post-stroke to enhance neuroplasticity, health, and well-being [3], lower the risk of recurrent stroke [4], and minimize the secondary conditions [5]. However, despite the known benefits of PA, many male stroke survivors find it challenging to meet the recommended guidelines to receive the health benefits of PA because they experience personal, social, and environmental barriers [6]. Experts in men’s health highlight that men, in general, place less importance on their health [7,8,9,10] and are therefore less likely to engage in activities that promote healthy lifestyles [11]. The collective impact of these barriers negatively affects their participation in PA. Their cardiovascular fitness and endurance decreases, leading to further inactivity and declining physical and psychological health (particularly self-efficacy) [3,12].

Stroke survivor self-efficacy is the most reliable predictor of PA participation [13]. Support from partners is an essential ingredient to enhance stroke survivor self-efficacy for PA following discharge from inpatient rehabilitation [14,15,16,17,18]. Partners of stroke survivors are recognized as being more influential than health professionals in supporting lifestyle behavior change [19]. However, the sudden and unexpected changes experienced after a stroke can leave partners of stroke survivors feeling overwhelmed with the additional responsibilities that they are expected to undertake [20,21,22,23,24,25,26,27,28,29,30,31]. The partner’s personal attitudes and beliefs about PA can also positively or negatively affect stroke survivor PA participation [14,19,32]. For example, if a partner perceives PA to pose a safety risk for the stroke survivor, they can become over-protective and hinder participation [33]. Factors that enhance a partner’s ability to successfully support their stroke survivor include gaining confidence and self-efficacy in the caring role, recognizing that some good can come from caregiving, having time for themselves, and being supported by other family members and health professionals [20,23,26,27,28,30,31,34,35,36,37,38].

Stroke survivors and their partners can feel anxious and vulnerable when transitioning from inpatient rehabilitation to home because of less access to health professional support [19]. Some partners report feeling abandoned by health professionals after discharge [28,30,31,34,38]. Several studies have shown that the partner’s information and education needs are unmet by health professionals prior to the patients’ discharge [20,24,26,27,28,29,30,35,37,39,40,41]. A lack of knowledge increases the partners’ feelings of anxiety, stress, and worry in their caregiving role [35,42]. While health professionals tend to provide information based on what they believe service users need [35], partners of stroke survivors are seeking evidence-based information and education that is individualized to their changing needs over time [19,23,27,28,31,39,40,41,43].

While many health professionals can prescribe PA advice, in New Zealand (NZ) (where this study was undertaken), physiotherapists are most likely to provide such education to stroke survivors and their partner. However, stroke survivors report a mismatch between physiotherapists PA advice and their personal aspirations [44]. For example, our previous work found that male stroke survivors were motivated by activities which could be incorporated into daily life, but physiotherapists provided exercises which they considered boring and meaningless [45]. In NZ, stroke survivors are also provided with written information about life after their stroke during in-patient rehabilitation [46,47]. Although these resources state that it is important to exercise, they contain very little information about why, how, or where to be physically active and they do not include information specifically for partners supporting their stroke survivor to engage in physical activity.

Considering that partners are essential to support stroke survivors to access and participate in PA [19,48], effective educational resources are sorely needed [49]. Such resources should include those that are tailored specifically towards partners and family members. A collaborative participatory co-design approach, where service providers and service users are valued equally for their knowledge and expertise, would ensure that the resources developed are meaningful and credible [49,50,51]. Co-created resources are more likely to be effective in enhancing PA self-management support [40,42,52,53] because they specifically target the needs of the service user [54].

The purpose of this study was to co-design an educational resource prototype to guide and empower female partners in providing self-management support for male stroke survivors’ participation in PA. The content of the resource prototype was informed by exploring female partners’ attitudes towards PA, the barriers, and the enablers for them to successfully support a stroke survivor’s PA, and what they would consider to be essential information to include in the resource.

## 2. Materials and Methods

### 2.1. Study Design

We used a participatory action research (PAR) methodology for this study.

### 2.2. Research Team and Reflexivity

The research team consisted of a doctoral student and two experienced academic research supervisors with expertise in qualitative and quantitative methodologies. All three researchers were female physiotherapists: two with clinical expertise in neurological rehabilitation and one in musculoskeletal sports physiotherapy. We engaged in the reflexivity process prior to and throughout the study using a reflexive diary and peer debriefing to ascertain and acknowledge our assumptions and potential biases in undertaking this research.

### 2.3. Recruitment and Participants

To recruit female partner participants (now to be referred to as partners or participants), we first sought community dwelling adult male stroke survivors > 6 months post-stroke, living in Canterbury, NZ. Male stroke survivors were recruited via health professional colleagues, the local Stroke Foundation organization, a fitness facility specifically for people with a disability, and via an advertisement placed in a free local community newspaper.

Interested male stroke survivors contacted the researcher directly or provided consent for their contact details to be passed on to the research team. The primary researcher (AC) met with interested male stroke survivors face-to-face in their home or a quiet place in the community to explain the study in more detail, answer any questions, and obtain written consent to complete a short questionnaire which included demographic information and levels of PA. Thirteen male stroke survivors completed the questionnaire (20 min duration). They were aged 53–82 years (mean 69 years) and 1.5–15 years post-stroke (median 4 years; mean 4.5 years). Twelve stroke survivors identified as NZ European and one as Māori. Using an adapted version of the trans-theoretical model of behavior change [55], eight stroke survivors were classified as regularly physically active and five were not regularly physically active. We felt it was important to determine the stroke survivor’s regularity of PA because their partners may face different challenges when supporting stroke survivors who were physically active compared to those that were not.

To recruit participants, we invited the 13 male stroke survivors to explain the study to their partner. We provided the male stroke survivors with an information sheet, which they could use to assist them in explaining the study to their partner. The male stroke survivors provided the contact details of their partners to the research team. The primary researcher (AC) contacted the interested partners (via telephone) to answer any questions and formally invite them to participate in the study.

### 2.4. Data Collection Procedures and Data Analysis

**Figure 1:**
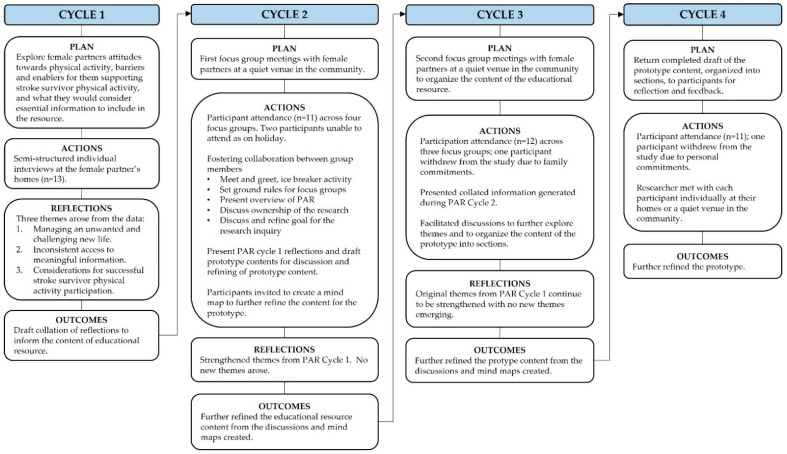
Participatory action research (PAR) cycles of planning, action, and reflection.

The data were collected across four PAR cycles of planning, action, and reflection using semi-structured individual interviews and focus group meetings. This data informed the content of the educational resource prototype. We describe the general data collection and analysis procedures which occurred across all the PAR cycles and then provide further detail of each individual PAR cycle in Figure 1 and Appendix S1 in the accompanying Supplementary Material.

The semi-structured individual interviews took place at each participant’s home, and focus group meetings were undertaken at a quiet venue in the community (e.g., at a local library). All were approximately 30 min to 1.5 h in duration. The semi-structured interviews and focus groups were audio-recorded then transcribed verbatim by a paid transcriber. We collected the partners’ written notes from the focus group meetings, and we kept a reflexive diary throughout the duration of the study.

We analyzed all the data (i.e., the transcribed individual interview and focus group data, the participants’ written notes, and reflective diary notes) for themes at each PAR cycle, using the general inductive approach described by Thomas [56]. To make sense of the raw data, one researcher (AC) read the entire data set to become familiar with the content of the transcripts and assigned codes to meaningful phrases within the text. Next, AC built a coding template to organize the codes into categories, sub-themes, and themes with accompanying descriptions using the scissor and sort technique [57]. Two other members of the research team (GS and HM) then independently crosschecked the coded data, themes, and descriptions. To do this, AC provided each independent coder with a clean version of the raw text along with the coding template. The independent coders matched the themes from the coding template with the sections of the text. The research team met at regular intervals during the PAR cycles to discuss and refine the codes, themes, and accompanying descriptions. An example of a coding template for one theme (theme 3) is included in Appendix S2. The analyzed data were presented back to the support person participants at subsequent meetings for reflection to ensure its accuracy and to provide a catalyst for further discussions.

### 2.5. Ethics

This study was granted ethical approval from the University of Otago Human Ethics Committee (Health) (reference H14/122).

## 3. Results

Thirteen partners, aged between 51 and 80 years (mean 65 years), provided written consent to participate in this study. They identified as NZ European (n = 11), Māori (n = 1), and Chinese (n = 1). According to the transtheoretical model of behavior change, 11 were classified as regularly physically active and two were not. The partners acknowledged that PA was beneficial for their personal health and well-being and participated in a broad range of activities (e.g., gardening, walking, running, swimming, household chores, and yoga). We used the modified Rankin Scale (MRS) and the questions from the MRS structured interview (MRS-SI) questionnaire to determine the level of support the partners provided for their male stroke survivors. Two stroke survivors were categorized with a slight disability, eight with a moderate disability, and three with moderately severe disability. Regardless of the level of disability, all the female partners came into this study as they felt they had a role to play in supporting their stroke survivor to participate in PA.

The findings represent the cumulated data collection and analysis from the four PAR cycles. The data were saturated after PAR Cycle 2 as no new themes arose. Three themes were reflected in the data, (1) managing an unwanted and challenging “new life”, (2) inconsistent access to meaningful information, and (3) considerations for successful PA participation for stroke survivors. These findings informed the content for the educational resource prototype (Appendix S3). The participants intent for co-designing the educational resource was to provide useful, informative, and motivational information for partners and family members supporting male stroke survivors’ PA participation.

### 3.1. Theme 1: Managing an Unwanted and Challenging “New Life”

The lives of the stroke survivors and their primary support persons were tipped off balance and, in many instances, the stroke survivor became egocentric and demanding. The participants realized there was no alternative but to take on the family roles of the stroke survivor in addition to their own. They felt utterly unprepared, frustrated, stressed, exhausted, and overwhelmed to manage these unwanted challenges and multiple additional roles. However, the participants identified that they themselves had changed, becoming more assertive because they had been forced to make family decisions alone.

*I’m trying to be all things. I’m not just being the housekeeper, housewife, I’m being the plumber, the painter, the lawn mower, the vege gardener, the taxi driver. The extra world is so demanding, unrelenting….it’s like running a train station. I was totally unprepared for it, and it was unrelenting, you never get a day off, you just can’t get out of it. You are absolutely out on your own….and you have got all the stress of this person…you are alone. (Participant 7, Individual Interview).*

Although the participants acknowledged the importance of PA, they also described frustrations and challenges to managing and maintaining their personal social, mental, and physical well-being. Attending to their physical health was particularly difficult because they prioritized their stroke survivor’s needs above their own. Cost and time were additional barriers to accessing PA. They also felt they had lost their freedom, spontaneity, and sometimes their friends. Social activities were a rarity and were often undertaken alone, with family, or with other stroke survivors because such people had a better understanding of the post-stroke challenges. Guilt was uppermost in the partner’s thoughts if they undertook activities to enhance their social and mental wellbeing. The participants recognized that they needed to accept their “new life” so they could preserve their mental wellbeing. They provided several ideas for enhancing the wellbeing of the support person to include in the resource. For example, acknowledging that life has also changed for the support person, taking time away from the stroke survivor without feeling guilty, and not being afraid to ask for help. Engaging support from others, such as family members, friends, neighbors, work colleagues, members of the stroke community, and health professionals, was valuable in helping them cope with their “new life”.

*Sometimes I feel guilty, like I’ve just started going to dance group again. It’s on a Monday night after work but I feel really bad because [husband] has been at home all day by himself. He wants me to do it and I do it, but I wish I could feel just that freedom…like in the past when he went to golf, and I went and did whatever [I wanted]. (Participant 2, Focus Group A-4).*

### 3.2. Theme 2: Inconsistent Access to Meaningful Information

Accessing meaningful information, including about health, was perceived by the participants as lacking or inconsistent. If information was provided, it was often not individualized for each stroke survivor, or varied substantially between health professionals, resulting in confusing mixed messages.

*We get mixed messages. People [health professionals] say, “Oh you must walk more” and then yesterday the physio came, and she said, “Don’t overdo it.” (Participant 13, Individual Interview).*

At times, the participants were overwhelmed with too much information. For example, one participant explained that she was so overwhelmed with written information she threw it in the rubbish bin unread. As such, the participants felt it important that the resource content included a preface which explained the intent of the resource, the target audience, why the guide was needed, and a suggestion to read only the aspects that were most important to the support person and the stroke survivor.

When considering access to information specifically about PA, the participants asked the primary researcher, a physiotherapist, to narrate this section of the resource, recognizing this was her area of expertise. The participants expressed that the PA content should include the benefits of PA, how much PA was required, how safe PA is, the types of physical activities that would be appropriate for stroke survivors, and suitable places to engage in PA.

The participants had varying opinions about the ideal time for its delivery. Most participants felt education about PA should occur when the stroke survivor was being discharged from rehabilitation and transitioning into the community. They expected that PA information and education should come from the physiotherapist or general practitioner. However, feeling frustrated by the inconsistent approach to information delivery from health professionals, the participants recognized that they needed to be proactive and educate themselves. They could do this at their own pace instead of feeling overwhelmed with information when they were not ready. Their learning came from a variety of sources such as their own self-directed researching (e.g., searching the internet or reading books from the library), listening to strategies from other support people of stroke survivors, and practical experiential learning through trial and error. The participants shared this knowledge in the educational resource included in the Supplementary Information (Appendix S3).

*Focus Group B-2 discussion*

*Participant 3: You’ve got to ask questions and keep asking questions if you don’t know.*

*Participant 8: But there’s no one really to ask. They [medical team] never took me to a room, it was just told to me [about his stroke] in the hallway, amongst all these other people. I actually got more information from getting a few books. That was the best thing.*

### 3.3. Theme 3: Considerations for Successful PA Participation for Stroke Survivors

The participants discussed the importance of encouraging their stroke survivors to engage in PA so that they could participate in family life and/or their pre-stroke activities. They explained their own philosophy of PA, which they had developed completely independently of any education, advice, or support from health professionals. The participants shared their practical strategies for encouraging PA for their stroke survivors. They recognized that regular physical activities, that included a balance of physical, mental, spiritual, and social wellbeing, were meaningful and enjoyable, and therefore provided motivation for PA participation.

*[The physiotherapist] gave us a lovely list of exercises and realistically I think he [stroke survivor] did them twice. It’s about your home life and achieving it all. I knew what would click with his psyche and his determination factor. It was a mission [stroke survivor feeding his cattle] but it took him from walking to walking properly, because out in the paddock he had to learn to balance when he was putting this electric fence standard in, and he was walking on uneven ground. I also thought that his social aspect of life was very important to him, so it was a matter for both physical and socially integrating him…being part of people within and around him was very beneficial for his well-being. (Participant 4, Individual Interview).*

When supporting and encouraging PA for their stroke survivors, the participants considered the impact of the stroke survivor’s physiological impairments (e.g., fatigue, physical impairments, or communication), psychological wellbeing, and other medical issues (e.g., pre-existing conditions or side effects of medication).

*One of the other challenges is [husband’s] tiredness. So you can’t force him out of bed if he can’t cope. You’ve got to take that into account how they’re feeling because if he just says he’s too tired, well…you have to go with it. (Participant 6, Individual Interview).*

The participants also explained that they worried about their stroke survivor’s safety when encouraging participation in PA. They understood that there were several risks, particularly when their stroke survivor had cognitive and/or physical impairments.

*He [husband] had just started walking out in the garden and getting out of the wheelchair. It had snowed and I had to go back to work. I said, “Whatever you do there is going to be more snow so don’t go outside, stay inside, don’t go outside.” He decided to go and look at the trees and plants [while I was out] and I drove up the drive and there he was stuck by the clothesline, and I don’t know how long he had been there for, and he wasn’t that well-dressed. His planning wasn’t good. Yeah, I get worried. (Participant 8, Individual Interview).*

One of the biggest barriers the partners encountered to supporting PA was the inaccessibility of the built environment. Therefore, prior to their stroke survivor engaging in physical activities in community settings, the participants scrutinized the environment very carefully to ensure it was accessible and safe.

*Focus Group B-2 discussion*

*Participant 11: Like [Participant 9], every time she takes her husband out [into the community], she goes there first and checks out the access.*

*Participant 4: We had a prime example of that on Thursday night where I didn’t [check accessibility prior] and some friends took us for a drink at [restaurant]….and it was so unsuitable for my partner….there was a lack of chairs and cobbled concrete to get in. The stools were all the wrong height….you know I should have gone to have a look.*

*Participant 8: You would have been on edge the whole time I bet.*

Alongside their philosophy of PA, the partners acknowledged that the successful participation in PA for their stroke survivor was highly dependent upon the provision of external support from themselves or others (i.e., family members and friends). They had adopted strategies such as providing positive reinforcement and incentives for their stroke survivor to participate in PA. However, the partners explained there was a very delicate balance between “telling” and “asking” their stroke survivor to engage in PA.

*When he starts snapping, “You haven’t had a stroke.” I think I have to back off a wee bit. I just have to take that on the cheek if he’s going to say that to me. I have to let it go over my head. But if he says he’s not going to do it he won’t do it. End of story. There’s nothing else I can do. I’ve done lots of things. I’ve said, “Look if you walk every day, just around the block, we’ll go out to lunch. So, I used to give him incentives to do it…but there is a fine line. (Participant 12, Individual Interview).*

The participants provided useful strategies for inclusion in the resource regarding motivating the stroke survivor to engage in PA. For example, involving extended family and friends and ensuring that health professionals were inclusive of the stroke survivor and support person in setting meaningful PA goals.

## 4. Discussion

This PAR study aimed to co-design an educational resource prototype to guide and empower female partners in providing self-management support for male stroke survivors’ participation in PA. The value of co-designing educational resources was illustrated in our study where the content drew on the collaborative expertise of the physiotherapist researcher and the partners. For example, while the participants provided meaningful ideas and strategies for PA from their experiential and self-directed learning, they acknowledged that the credibility of the resource would be enhanced if the PA section of the resource was written by the physiotherapist researcher with expertise in the management of people living with neurological conditions.

The participants in our study recognized that undertaking PA is essential for stroke survivors to return to meaningful activities within the family unit and to develop social participation. They intuitively and knowledgeably created their own philosophy of PA to support their male stroke survivors by finding or creating activities that encompassed spiritual, mental, physical, and social wellbeing. The participants were frustrated with the inadequate or confusing information provided by health professionals which led them to look for information through their own self-directed research, word of mouth (from other partners), or by a trial-and-error approach. A small but growing body of evidence supports our findings that caregivers of stroke survivors are seeking information from a variety of sources other than health professionals [26,28,30,35,37,39,41]. As family members’ beliefs and behaviors are strongly influenced by the information they can access [19], it is essential that health professionals provide meaningful, individualized information to both the stroke survivor and partner [12,58].

Health professionals need to pay careful attention to the timing to deliver information. The participants in our study felt the best timing for information and education about PA was in the transition phase of discharge from inpatient rehabilitation into the community. Both written and verbal delivery is recommended [19,24,43,53,58]. Furthermore, the partners of stroke survivors welcome information and education from those who share similar personal experiences (i.e., other partners of stroke survivors) because they are perceived to hold meaningful, robust, credible, and authentic evidence [19,39]. This strengthens the value of developing educational resources using participatory co-design methods.

While most of the content of the educational resource prototype focused on ways to support the stroke survivor to participate in PA, the participants in our study were adamant that strategies which specifically addressed the partner’s self-management be included. Although the participants prioritized their stroke survivors’ needs before their own, they recognized the importance of attending to their own health (particularly their mental and physical wellbeing), so they could build confidence, self-efficacy, and resilience for their support role. The content of the educational resource provides several meaningful and credible strategies to empower the partner’s self-management. These findings resonate with other research, which examined the partners’ experiences of coping and adapting to life after stroke [28,41,59]. If partners feel in control and able to cope, it is likely that the stroke survivors’ participation and subsequent outcomes will be enhanced. However, partners require targeted support from the health system [60] to empower them to feel confident and self-efficacious in their support role [13,21,23,35,59,60,61,62]. As health professionals rely on partners to provide self-management support after discharge from rehabilitation [63,64,65], an inclusive and collaborative person- and family-centered approach where partners are considered as an equal and valued member of the team alongside the stroke survivor is recommended [12,27,29,59,61,63,66].

While there is a growing body of evidence using co-design methods with informal caregivers of people with long-term conditions, to our knowledge, there are no studies which collaborate with the partners or family members of stroke survivors. Furthermore, there are no such studies from the NZ context, hence our study is novel in this regard. One of the limitations of the co-design literature is the paucity of evidence that evaluates the participants’ reflections of the co-design process as most report on the study’s outputs. Therefore, it is challenging to provide recommendations for future research and practice. Stroke survivors and people with multiple sclerosis who have collaborated in co-designing educational health resources report feeling empowered because their voices are prioritized and valued, and acquire a sense of accomplishment that the output produced may benefit others [39,67]. While it is possible that these reflections could be similar for caregivers, careful consideration should be given to the additional burden of the prolonged engagement required for co-design for this already overwhelmed population. Kuluski et al. [68] recommends that researchers and health professionals collaborating with caregivers develop flexible and creative ways to lessen this burden. For example, in our study, for each PAR cycle, we created multiple focus groups across multiple easily accessible locations to accommodate the participants’ busy schedules. 

This study has limitations. The findings are primarily limited to NZ European, female partners as support people of male stroke survivors in heterosexual relationships. People whose gender identity and/or sexual orientation or ethno-cultural identity differs from the participants in this study may report different experiences. For example, Larson et al. [25] indicated that female partners of male stroke survivors experienced poorer psychological health than their male counterparts in heterosexual relationships. A small body of evidence also reports that male partners in heterosexual relationships are more likely to be given advice about alternative care options than their female counterparts [25,28,41]. It is also possible that sampling bias is present. The partners all had strong beliefs and attitudes about the importance of PA for their stroke survivors and for themselves, which may mean they are more likely to offer to participate in research about PA. The findings may be different for support people with alternative views about PA. Therefore, prior to implementation, it is crucial that the educational resource prototype developed in our study undergoes acceptability and usability testing with a wider audience of support persons. Including support persons that represent gender diversity, ethnocultural diversity, and other family members and friends would enhance the accessibility, relevance, and credibility of the educational resource.

## 5. Conclusions

This study aimed to co-design an educational resource prototype to guide and empower female partners in providing self-management support for male stroke survivors participation in PA. The content of the resource was informed by the participants’ lived experiences of supporting their male stroke survivors to participate in PA. The participants provided strategies to manage their unwanted and challenging new life, ideas for managing inconsistent access to meaningful PA information, and considerations for the successful participation of the stroke survivor in PA.

Co-design primarily prioritized the voice and expertise of the participants to create a resource that is meaningful for support people of male stroke survivors. However, the physiotherapist researcher’s expertise and knowledge of PA was also valued. The findings in our study suggest health professionals consider the value of collaborating with support people of stroke survivors through a participatory person- and family-centered care approach.

Further, evaluative research is recommended to explore the acceptability and usability of the educational resource and the participant’s reflections of participating in the co-design process. This would provide useful suggestions for health professionals co-designing educational health resources in the future.

## Data Availability

Appendix S2 provides an example of how the raw data supported the results. The raw data are not publicly available. De-identified data can be sought from the corresponding author for the purpose of research studies that hold appropriate ethics approval.

## Supplementary Materials

The following supporting information can be downloaded at: https://www.mdpi.com/article/10.3390/ijerph192416856/s1, Appendix S1: Participatory action research (PAR) cycle additional details; Appendix S2: Example of the coding template for Theme 3; Appendix S3: Educational resource prototype.
