# Supplementary material for: Co-Design of an Educational Resource with Female Partners of Male Stroke Survivors to Support Physical Activity Participation
Source: Int J Environ Res Public Health. 2022 Dec 15;19(24):16856. doi: 10.3390/ijerph192416856 (PMC9779113; doi:10.3390/ijerph192416856)
Supplement: Supplementary file 1 [file ijerph-19-16856-s001.zip › Appendix S1 PAR cycle details.pdf]

Appendix S1. Participatory action research (PAR) cycle additional details

PAR Cycle 1

In the first PAR cycle, support persons engaged in a semi-structured individual interview to explore 1) their beliefs about PA for themselves and for stroke survivors, 2) factors that the support persons perceived would help or hinder them to empower their stroke survivor to be physically active, and 3) information that would be meaningful to be included in an educational health resource (Table A).

Table A. Semi-structured interview guide with support person participants

Research Questions	Semi-structured interview guide questions
What are support person's understandings and beliefs regarding PA in general?	How would you define PA?<br>What kinds of activities would you be doing if you were physically active?<br>What are your beliefs about PA or exercise?<br>What motivates you to be physically active?<br>Are there any challenges for you to be physically active?
What are support person's understanding and beliefs regarding PA following stroke?	What do you understand about PA following stroke?<br>Where did you come by this knowledge?<br>Is there anyone in particular that you believe should provide education about PA following stroke?<br>Is there anyone in particular that you believe should support stroke survivors to be physically active?<br>When along the stroke recovery continuum would be the ideal time for education about PA to occur (acute setting, inpatient rehabilitation, community setting after discharge)?
What factors help or hinder support persons of male stroke survivors to support their stroke survivor to be physically active?	Can you share any experiences where your stroke survivor has been physically active after discharge from the hospital?<br>Did you play a role in assisting with this PA? Can you tell me about this?<br>Can you think of any factors that help you to support your stroke survivor to be physically active? What are these?<br>Can you think of any factors that have hindered or challenged you to support your stroke survivor to be physically active? What were these?
What do support persons of male stroke survivors perceive as useful	If you were to put together a resource specifically for support persons of male stroke survivors to support

Research Questions	Semi-structured interview guide questions
information to be included in an educational health resource?	stroke survivors to be physically active, what would you include?

Abbreviations: physical activity (PA)

PAR Cycle 2

The support person participants attended a focus group meeting to share their knowledge and experiences on the topic of inquiry. It was difficult to find a day and time suitable for all PAR group members, therefore there were four focus groups within this PAR cycle. Five participants attended the first focus group and two participants each attended the other three focus group meetings. Two participants were unable to attend because they were on holiday. The aim of the focus group meetings was to foster collaboration between the group members. This involved an icebreaker activity so the participants could get to know each other, setting of the ground rules, discussing ownership of the research, and reviewing the goal for the study. At this meeting, the participants were provided with a hard copy summary of the emerging themes, sub-themes, and descriptions, along with the participants initial ideas for the educational resource content from PAR Cycle 1. Using the draft hard copy of the themes and our discussions and reflections at this focus group meeting, the participants were invited to construct a "mind map" to further refine further the content for the educational resource.

PAR Cycle 3

The plan for the third PAR cycle was to organise the content of the educational resource into sections. The participants attended a second focus group meeting where the researcher presented the revised collated data from PAR Cycle 2 and facilitated discussions to refine and group the educational resource content. Like PAR Cycle 2, to accommodate the support persons busy schedules, three separate focus group meetings were organised. One participant withdrew from the study due to family commitments.

PAR Cycle 4

The researcher emailed the completed educational resource content from discussions in PAR Cycle 3 to the participants to inquire whether it reflected their thoughts and experiences accurately. Only one participant provided written feedback therefore, we contacted the remaining 11 participants and invited them to engage in one final individual meeting to review the educational resource prototype. Ten participants met with the researcher and the final participant withdrew from the study due to personal commitments. The researcher then collated the participants feedback and drafted a first prototype for the resource.
