# Supplementary material for: Co-Design of an Educational Resource with Female Partners of Male Stroke Survivors to Support Physical Activity Participation
Source: Int J Environ Res Public Health. 2022 Dec 15;19(24):16856. doi: 10.3390/ijerph192416856 (PMC9779113; doi:10.3390/ijerph192416856)
Supplement: Supplementary file 1 [file ijerph-19-16856-s001.zip › Appendix S2 Coding template example.pdf]

Appendix S2. Example of the coding template for Theme 3

Theme 3. Considerations for successful stroke survivor physical activity participation

Codes	Participants	Categories	Descriptions	Sub-theme
FP fitness	7, 8, 11			
Improve FP's health	1, 2, 6, 8-10, 12, 13	Benefits of PA for FP well-being		
FP mental well-being	1, 2, 4, 6, 8, 12			
FP physical well-being	1, 2, 4, 6, 7, 10, 11			
SS FPiritual health	4, 13			
SS physical well-being	1, 4, 7, 13	Factors for successful participation in PA for SS		
SS mental well-being	1, 4, 9, 13			
SS social health	4, 5, 10, 13			
FP tries to find a variety of activities as these are important to motivate SSs to be physically active	2, 4, 6, 8	Variety	The FPs understood that PA was beneficial for their own personal health and well-being so they could support the SSs needs. For successful participation in PA, the FPs identified that PA needs to incorporate a balance of physical, mental, social and FPiritual well-being. Activities should therefore be meaningful, enjoyable, participatory, and socially integrated.	
PA needs to be regular/routine	2,4, 5, 9, 13, FGB2	Routine		
PA needs to be social	1, 4, 5, FGA1FGB2, FGB3,			
Going on outings that include PA	5			A philosophy for PA
Social well-being important to SS recovery, morale, confidence, mental well-being	1, 3, 4, 8, FGA1, FGB2, FGB3	Social integration		
Social integration helps SS to think about others rather than dwell on impairments	4			
Male bonding is important post stroke	8			
PA in a group of like-minded individuals is good for social integration and motivation	4, 8, 10, 13, FGB2			
FP works out different ways to approach community outings	FGA4			
PA needs to be enjoyable/pleasurable	2, 4, 5, FGB2	Meaningful and enjoyable		
PA needs to be meaningful to the SS	4, 10, 12, FGA4, FGB2			
PA need to be participatory and contribute to life roles and pre-stroke activities	1-5, 7, 10-12, FGA3, FGB3	Participatory		
Creating competition is important	5	Competitive		
PA needs to be challenging but target the SSs level of disability	4, 6, 8	Challenging		
Stroke related impairments (fatigue, physical impairments, communication) affects participation in physical activity	2, 3. 4, 6, 9-12, FGA3, FGA4,	Stroke related impairments	The FPs considered the impact of their SS's physiological and psychological impairments when encouraging physical activity. They worried about physical safety particularly when the SS had physical and/or cognitive impairments that prevented them	
Mental well-being (fear of falling, frustration, motivation, self-drive, depression) affects engagement in physical activity	1, 2, 4, 6, 8-10, 12, FGB3			Consideration of the SS and the environment
FPs develop a pragmatic approach to managing SS mental well-being (e.g., suicidal thoughts)	FGB3	Mental well-being		
FP warned about SS becoming depressed so encouraged pre-stroke activities post stroke	8			

Codes	Participants	Categories	Descriptions	Sub-theme
FP learns to read SS mood so understands when to leave him be	4		from making independent decisions or in being able to negotiate a challenging environment independently. Therefore, the built environment required careful scrutiny to ensure it was accessible and safe for the SS to use.	
FP needs to consider how the SS is feeling	FGA3, FGB3			
Other medical issues (e.g., side effects of medications, pre-existing conditions) affect ability to be physically active	6, 8, 9	Co-morbidities		
Facilities that are a long way from home are challenging to access	8, 13	Built environment accessibility		
PA is challenging when the built environment is inaccessible	4, 6, 9, 10, 13 FGA2, FGA3, FGB2			
Built environment access can be a barrier to social integration	FGA3			
Gyms that are accessible for people with disabilities are needed	4, 13			
Accessible built environment takes the stress out of the outing	FGA1			
FP problem solves herself to try and make access easier	6			
FP needs to check built environment prior to going on an outing with SS	4, 9, FGA1, FGA2, FGB2			
FP problem to accommodate SS impairments within changing environments for safety	4, FGB2	Safety considerations		
Safety is paramount when making PA participatory	4, FGB2			
FP things about, worries about, and advises about safety	3, 5, 8, FGA3, FGA2			
FP recognises that SSs sometimes don't recognise their limitations (e.g., due to cognitive impairment) which is a safety issue	3, 10, FGA2			
FP finds it challenging to manage safety	FGA2			
FP needs to trust SS in calculated risk-taking behaviour	FGB2			
FP forces SS to make decisions to take back control of their lives	6	Nagging vs nurturing	The FPs recognised that participation in PA for their SSs was more fruitful if they provided external motivation and encouragement. They also acknowledged that other people (e.g., family members and friends) were also helpful in encouraging participation in PA for the SS.	External motivation and support
FP forces/challenges/encourages SS to participate in PA and life roles	3- 6, 8, 10, 13, FGA1, FGA2, FGB2			
Tough love important to drive motivation for physical activity	1, 4, 12			
FP acts as SS conscience for PA by prompting	3, 9, 13			
FP recognises that she can only do so much – it's up to the SS in the end	1, FGB3			
How much can the FP push the SS to be physically active – need to find the balance	1, 12			
SS likely to say no to primary FP but pay more attention to others (e.g., family members)	FGA3, FGB3			
FP needs to be stubborn and not give in to SS otherwise goals are not achieved	9, 10, FGB3			
FP provides encouragement, support, and praise for challenging/non-routine tasks	8-12	Praise and encouragement		
FP recognises that although SSs are adults, they reFPond better when treated like children (i.e., reward system for motivation)	FGB3	Provide rewards		
Family members and friends	3, 4, 6, 8, 9, 11,12, FGA3, FGA4	Family members, friends, and neighbours		
Neighbours	8			
Members of community groups (e.g., church)	1			
Work colleagues	4, 8	Work colleagues		
Health professionals	4, 6-8, 10, 11, 13, FGA2	Health professionals		

Codes	Participants	Categories	Descriptions	Sub-theme
Gym staff	8			
Other FPs in the community is a great way to share problems	3, 6, 8, 9, FGA1, FGA4, FGB1, FGB2	Members of stroke community		
Other SSs	10, FGB2			
Recognition from FP not to burden your support networks as they too have stressors in their lives	9, FGB2	Care with overburdening		

Abbreviations: Female partner (FP), focus group (FG), physical activity (PA), stroke survivor (SS)
