# Supplementary material for: Co-Design of an Educational Resource with Female Partners of Male Stroke Survivors to Support Physical Activity Participation
Source: Int J Environ Res Public Health. 2022 Dec 15;19(24):16856. doi: 10.3390/ijerph192416856 (PMC9779113; doi:10.3390/ijerph192416856)
Supplement: Supplementary file 1 [file ijerph-19-16856-s001.zip › Appendix S3 Educational resource prototype v2.pdf]

The Carer's guide to the physical activity galaxy

A guide to supporting and encouraging male stroke survivors to participate in physical activity

An educational resource prototype co-designed by:
13 female partners of male stroke survivors
Dr Allyson Calder
Associate Professor Hilda Mulligan
Associate Professor Gisela Sole

Illustrated by: Mrs Marilyn Thorp

Canva

This prototype is directly linked and informed from the research publication titled, “Co-design of an educational resource with female partners of male stroke survivors to Support Physical Activity Participation.”

The prototype was co-designed by 13 female partner support persons of male stroke survivors and physiotherapist researchers: Dr Allyson Calder, Associate Professor Gisela Sole, and Associate Professor Hilda Mulligan from the School of Physiotherapy, University of Otago, New Zealand.

Illustrator: Mrs Marilyn Thorp

PLEASE NOTE: The intent of the prototype was to provide useful, informative, and motivational information for partners and family members supporting male stroke survivors’ PA participation. However, the prototype was created by a very small group of largely New Zealand European female partners and three physiotherapist researchers. Two researchers have clinical experience in neurological rehabilitation and one in musculoskeletal sports physiotherapy. Prior to using this resource, it is crucial that the educational resource prototype developed in our study undergoes acceptability and usability testing with a wider audience of support people. Including support people that represent gender diversity, ethnocultural diversity, and other family members and friends would enhance the accessibility, relevance, and credibility of the educational resource.

Corresponding author: Dr Ally Calder ally.calder@otago.ac.nz

Contents

	Pages
Preface	1
Physical Activity	2
Stroke Survivor Mood Management	8
Looking after your health and well-being	10
Notes	13

PROTOTYPE

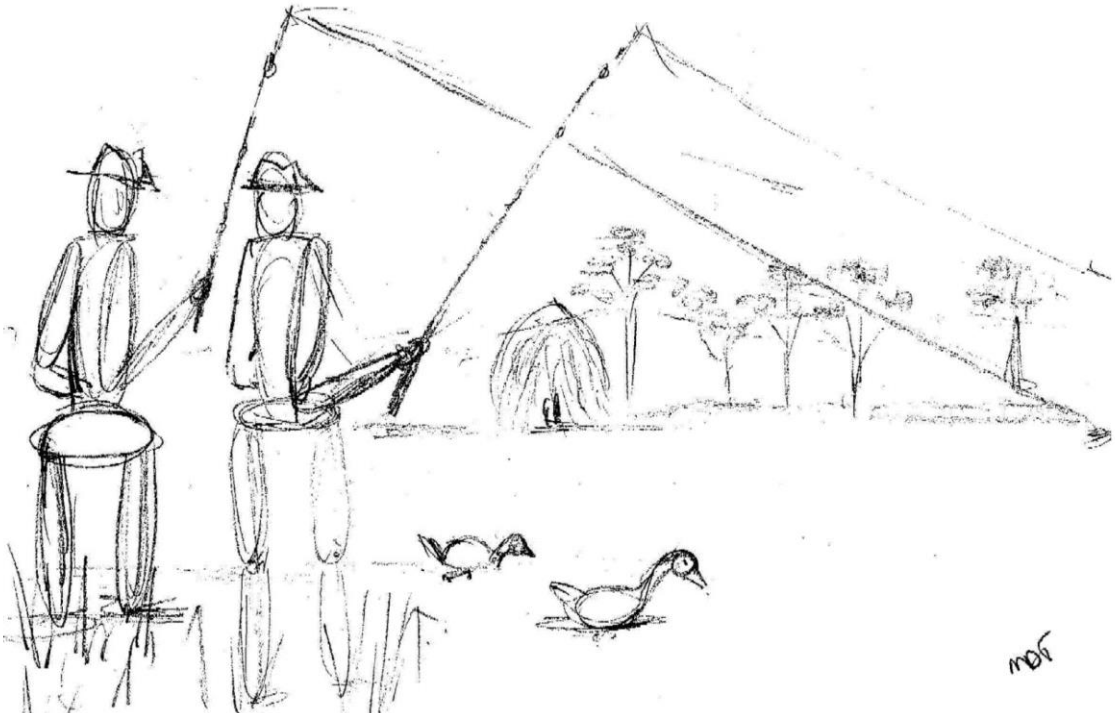

Preface

What is the intent of this guide?

- This guide provides ideas for encouraging stroke survivors to participate in physical activity.

Who is this guide targeted towards?

- The guide is targeted towards support persons of stroke survivors (i.e., family members and friends).

Why is this guide needed?

- It is beneficial for stroke survivors to engage and participate in physical activity (read more on this below)

How was this guide developed?

- It became apparent during research for a PhD thesis titled, "Access to physical activity for men following stroke" that those who support men after stroke have a large influence on their actions and decisions, including whether and how they are physically active.
- This guide is a result of female partners of male stroke survivors working together with the PhD student, a practicing physiotherapist and researcher. Their intent was to develop a guide that they know will be useful and informative, as well as motivational for the user.

About this guide

- This is a guide only. Every stroke survivor is unique and has different needs.
- You do not have to read the entire guide from cover to cover.

> "This guide provides a lot of information which can be overwhelming, so why not read the aspects that seem most important to you and your stroke survivor. You can come back to the rest of it when you feel you are ready."

PROTOTYPE

Physical Activity

What is physical activity?

Physical activity happens when you move the muscles of your body in a way that uses energy.

Why is physical activity important for stroke survivors?

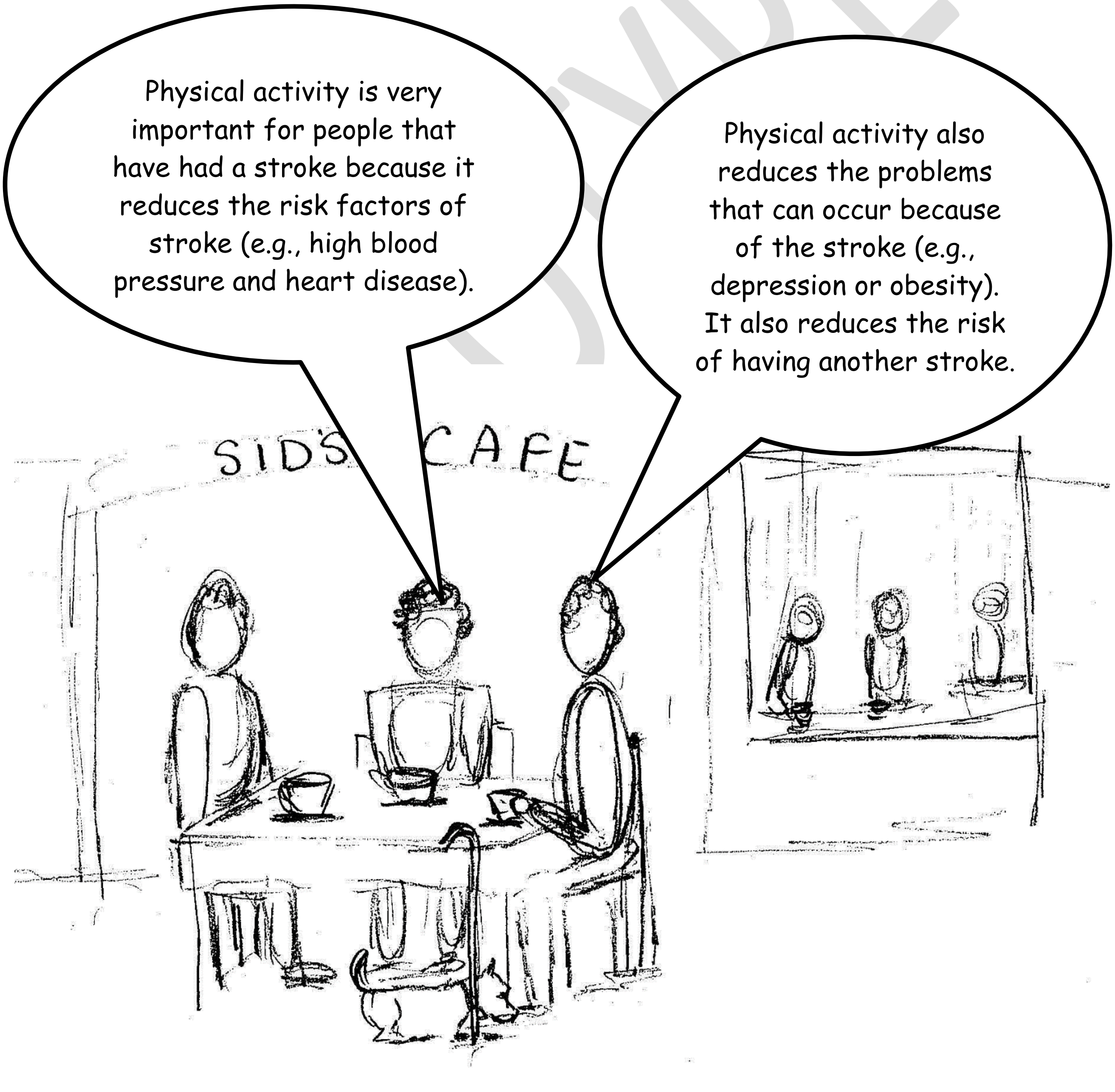

What types of physical activities are recommended?

Activities that work on…..

Fitness	Strength
Balance	Flexibility

Activities that provide a balance between spiritual, mental, social, and physical well-being are important. Support networks (e.g., family members and friends) are an important "anchor" often needed to help encourage the stroke survivor to be physically active.

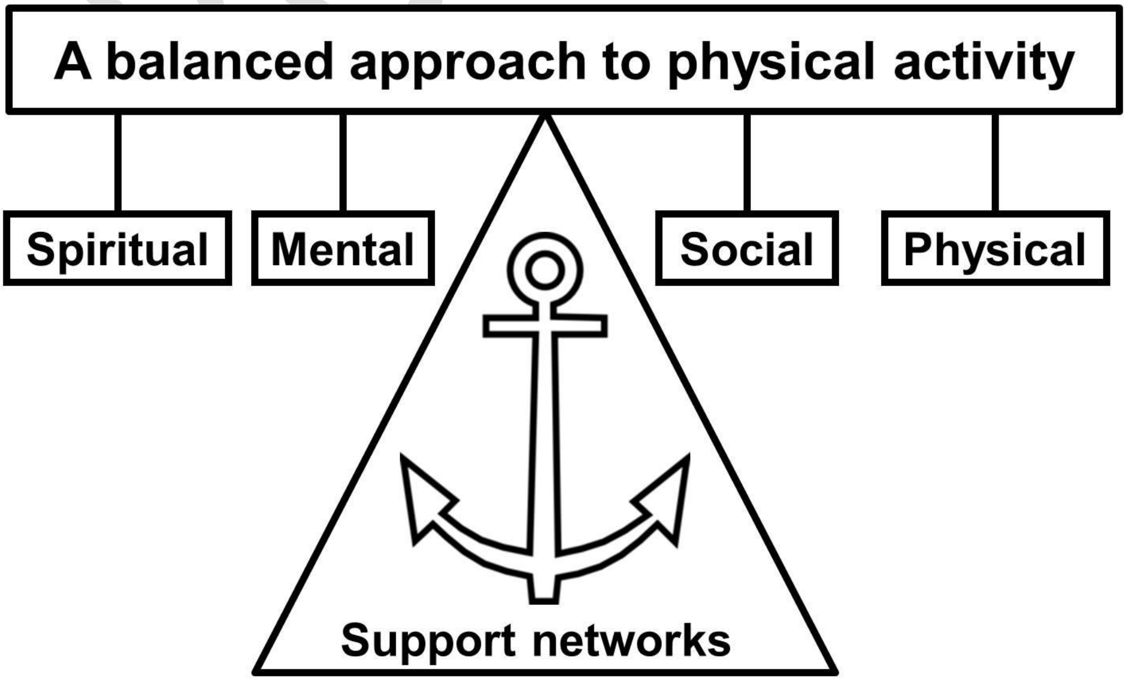

How safe is it to be physically active?

- Physical activity is beneficial for your health and well-being after stroke however, it is important that you are guided by your health professional (i.e., your General Practitioner (GP) or physiotherapist).
- If you are unsure how to engage in physical activity safely, it is important to keep asking your health professional until you have a clear understanding of what is safe.
- When considering participating in physical activity is important to consider the risk of injury (e.g., falling).

Physical activity guidelines

Everyone has different needs when it comes to physical activity. Be sure to ask your doctor or physiotherapist for further recommendations. As a general guide, moderate intensity physical activity is best after stroke.

From: https://www.heart.org/en/healthy-living/fitness-basics/aha-recs-for-physical-activity-in-adults

> "When done at a moderate intensity, your heart will beat faster, and you'll breathe harder than normal, but you'll still be able to talk. Think of it as a medium or moderate amount of effort."

Finding the right physical activities for your stroke survivor

- Stroke survivors are more likely to engage in physical activity if it is enjoyable, meaningful, fits in with daily tasks, or was something they used to do before the stroke.
- It does not have to be the same activity every day – remember variety is the spice of life!

"Sometimes finding activities that you can do together that are social are great (e.g., going to a café or the movies with family and friends, or playing with your grandchildren). You could park the car some distance away and walk to the venue."

"Joining other groups that include physical activity (e.g., your local stroke group) can be a good way to get started. Make sure the exercises are pitched at the right level for your stroke survivor."

"If your stroke survivor has problems with communication, do not let this hold back attendance at such an activity. You may simply need to explain to those offering the activity how to best communicate with your family member."

If you are stuck deciding what the right activity might be, consult your physiotherapist or GP. Make sure you are all on the same page with what you want to do and work together as a team to make this happen.

"Remember, you know the stroke survivor best."

Financial support

- Your family income may be less if the stroke survivor and/or the support person is unable to work. You may need to decide on priorities for spending.
- Talk to the social worker or GP if you feel you need some financial support so that your stroke survivor can engage in physical activity.
- Work and Income New Zealand (WINZ) can sometimes help with the cost of fitness centre fees and taxi fares.

"You have to ask because they will not tell you."

Take the following factors into account…..

- The stroke survivor's stroke related problems – fatigue, weakness, communication, and cognition.
- The stroke survivor's mental well-being (i.e., mood and motivation).
- Other medical conditions such as arthritis, pain etc

Consider the accessibility of the environment where the stroke survivor will be physically active…..

- Can you get into the building safely?
- Are there accessible toilets/bathrooms?
- What is the surface of the ground like? For example, if your stroke survivor is using a wheelchair; it can be difficult to push it over gravel and uneven ground.
- How far away do you have to park?

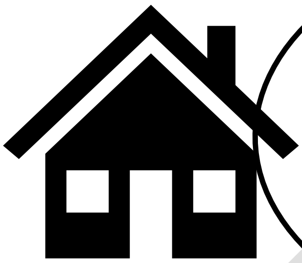

"Checking out the accessibility of the environment prior to accompanying the stroke survivor can save a lot of stress for both of you."

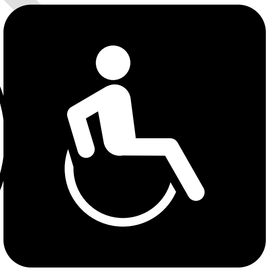

Become a researcher

- Learn as much as you can from others (e.g., other stroke survivors and their families, health professionals etc).
- Go to coffee groups for stroke survivors and their support persons (e.g., stroke carers group, stroke aphasia support persons group) as these provide support and information from others "in the know".

"Don't hesitate to ask everyone and anyone! You will always find at least one thing that is useful for you."

"Health professionals tell you the medical side of things but tend not to focus on the rest! You may think they will tell you everything you need to know but it is actually from networking with other support persons or stroke survivors (word of mouth) that you find the most informative and useful information ."

PROTOTYPE

Where to be physically active

There are many things you can do to be physically active.....here are a few ideas to get started:

Fitness centres (i.e., gyms and swimming pools). Gym instructors are very useful to help with ideas for physical activity. If you choose to swim, it is recommended the stroke survivor is accompanied by their support person. Find out how to support your stroke survivor in the pool from your physiotherapist or from staff at the pool.

Some rehabilitation facilities offer programmes specifically for people with stroke.

Household activities and social activities can incorporate physical activity (e.g., cleaning windows, doing the dishes, gardening, getting in and out of the car, going to a café or restaurant, going to the movies, shopping in a mall or supermarket).

Men's Shed is a great way to be physically active for male stroke survivors. Find out if there is one in your area.

Getting out to your local park or gardens.

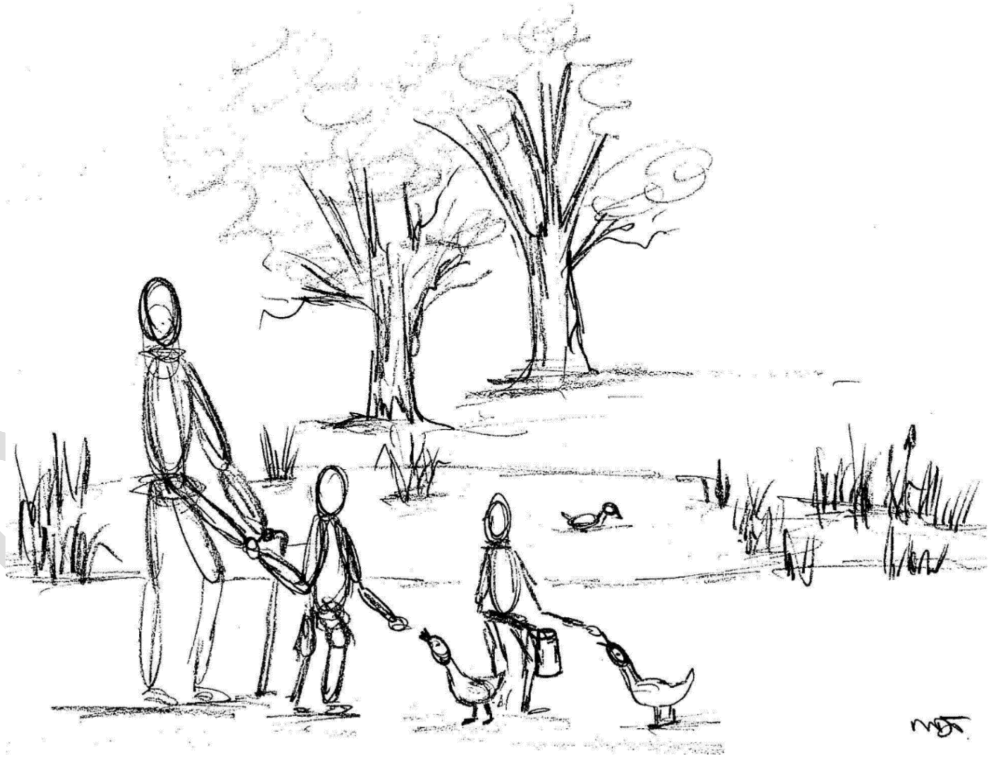

Look on your local council website as they often have information about local events.

Find out about the Green Prescription programme in your local area.

Depending on the level of physical activity achievable for the stroke survivor, attending competitive events can be challenging but rewarding.

Stroke Survivor Mood Management

A variety of moods and behaviours can occur in the stroke survivor, and this is normal. Here are some ideas for helping you cope with this should it arise and also help you to encourage physical activity:

For many reasons the stroke survivor may not be motivated to engage in physical activity. As the support person, you may be their main advocate and motivator.

"At the end of the day it is up to the stroke survivor to engage in physical activity.....you cannot force them to participate but you can encourage."

"It is an adjustment for both you and the stroke survivor. Consider the quality of your relationship when encouraging physical activity particularly when the stroke survivor's mood is low."

Some "top tips" for motivating your stroke survivor without becoming frustrated:

Get everyone involved as soon as you can – family members, friends, neighbours, health professionals etc. Often a motivational chat from others is more accepted by the stroke survivor rather than what might feel like a "lecture" from their spouse or partner.

The stroke survivor is more likely to participate in activities that are social, enjoyable, and meaningful. Offer choices.

"Remember we all need company and conversation."

Do not give up...keep motivating no matter how frustrated you get! Take into account how you both may be feeling. Acknowledge their mood and keep in mind that the stroke survivor's life has changed overnight just as yours has. Try to acknowledge all achievements no matter how small.

PROTOTYPE

"Ask your stroke survivor why they feel they are not able to be physically active. It may be they are fearful, fatigued, or frustrated. Remember it takes everyone time to adjust to the new situation."

"Think of incentives to encourage physical activity and share a reward together."

Explain the benefits of physical activity (see earlier in this booklet).

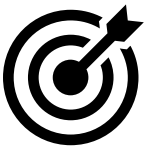

Sometimes setting goals (little challenges) and making a plan of how to achieve these can help.

"Be firm, confident, and encouraging."

"Humour can sometimes help to decrease your frustration. Do things together that make you laugh."

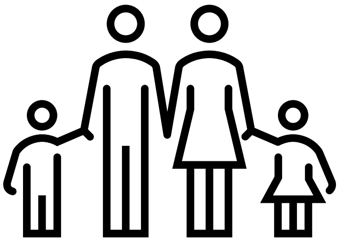

Consider your stroke survivor's sense of belonging and their purpose in the world. Maybe they could do some of the activities they used to do prior to the stroke in a modified way, which may make them feel valued and contributing to family life. Talk with your stroke survivor about the increase in your roles post-stroke and if they could assist with at least one job, how you would find that helpful. Volunteer work may also help your stroke survivor feel valued.

PROTOTYPE

Looking after your own health & well-being

It is important that you look after yourself and make this a priority. It is easy to let your well-being slide.

"Your time is limited caring for your stroke survivor, and you may find they become very demanding!"

"It's just another thing to do!"

"You may be tired, exhausted, unmotivated, overwhelmed, and stressed."

"You may find yourself with additional jobs that your spouse/partner used to do."

"You may not have the financial resources."

"You may feel guilty because you are leaving your stroke survivor alone or you may recognise your stroke survivor may not be able to join in the activities."

"You are likely to prioritise your needs below those of your stroke survivor."

These are normal feelings and responses for a support person of a stroke survivor.

It might not be easy but try to…..

Re-energise! Have time away (e.g., a holiday) or timeout (e.g., going shopping, coffee with friends on your own) for yourself without feeling guilty so you can continue to be your stroke survivor's main support person.

"With little time to yourself, it is easy to become exhausted, angry, frustrated, and annoyed with life."

Acknowledge that life has changed – this is a new normal, a new journey that you have to accept just as the stroke survivor does.

Try to find the balance in your health and well-being. Consider trying to incorporate all aspects of well-being in your life:

**Physical**	• Keep your strength so you feel confident managing your new role.<br>• Try to get lots of sleep.<br>• Take time out to exercise; a low cost option is walking or cycling.<br>• You may be able to exercise with your stroke survivor depending on their level of mobility.
**Spiritual**	• Work on your "inner spirit" and mindfulness.<br>• Counselling could be helpful.
**Mental**	• Feeling overwhelmed, stressed, guilty, frustrated, and angry are all normal responses for a support person following stroke.<br>• Take timeout for you.<br>• Counselling could also be helpful.<br>• Talk to your GP if you feel your mood is low.
**Social**	• Choose activities that are social as we often feel re-energised with company and conversation.

PROTOTYPE

"Anchor yourself firmly in your support networks if you can!"

"Don't be afraid to ask for help. This is not always easy. If your friends offer to help, take them up on it. If it is difficult to do this ask your friends not to give up on you. Sometimes you have to teach your friends what support would be useful for you."

"If you feel isolated or 'left behind' seek support (e.g., GP, health professionals, family members, and friends)."

"Don't be afraid to have a good cry. It doesn't mean you can't cope but it helps you to release pent up feelings!"

PROTOTYPE

Notes

PROTOTYPE

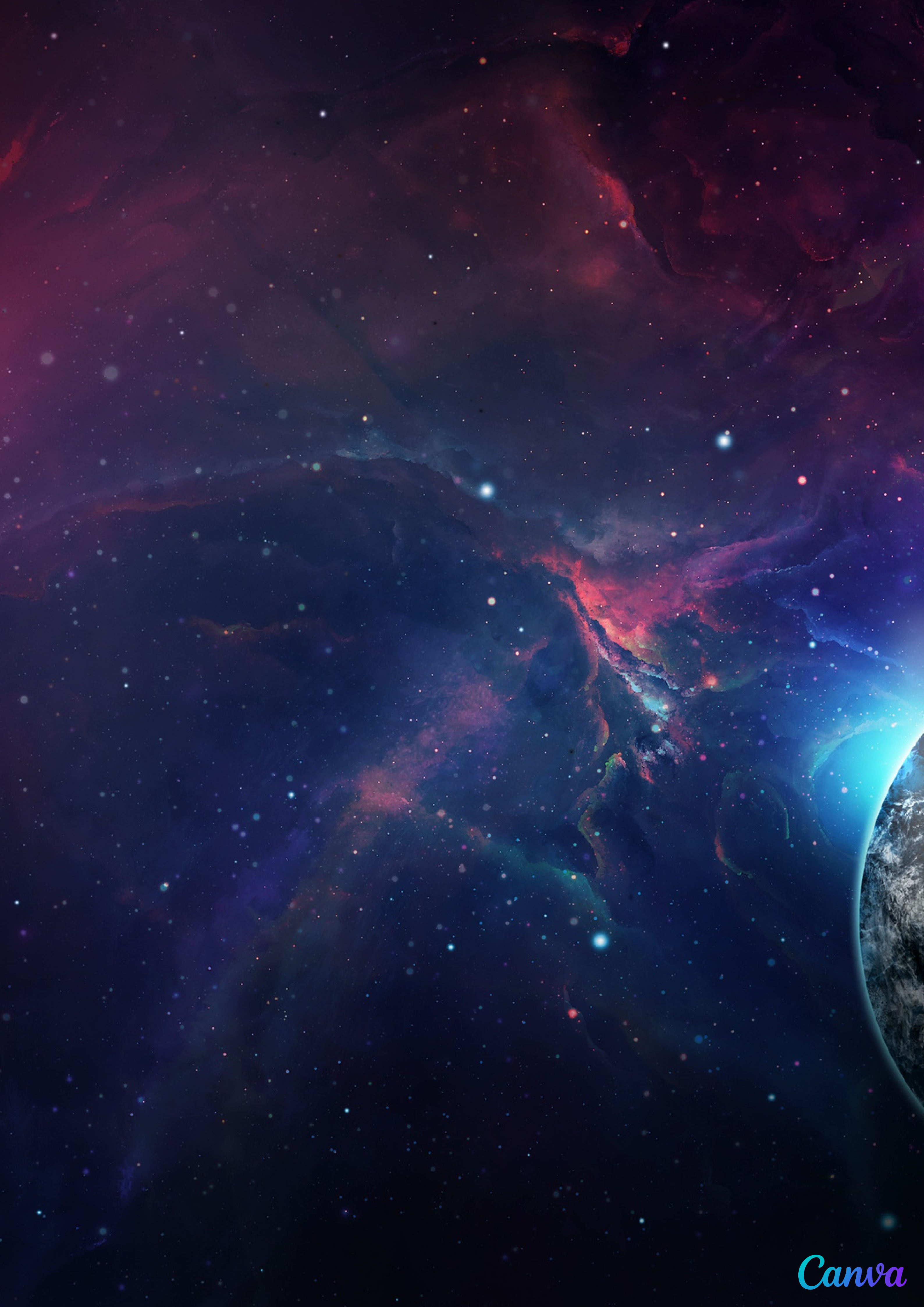
Canva
